# Supplementary material for: Predicting the animal hosts of coronaviruses from compositional biases of spike protein and whole genome sequences through machine learning
Source: PLoS Pathog. 2021 Apr 20;17(4):e1009149. doi: 10.1371/journal.ppat.1009149 (PMC8087038; doi:10.1371/journal.ppat.1009149)
Supplement: S1 Methods — (DOCX) [file ppat.1009149.s001.docx]

**S1 Methods**

Imbalance between outcome classes can bias machine learning algorithms and impact predictive performance [1,2]. As our data thinning approach (see main Materials and methods) had the additional secondary effect of reducing class imbalance between host categories, we compared this to alternative sampling methodologies intended for class imbalance correction: class weighting inversely proportional to prevalence of class observation; random downsampling of all classes to equal frequency of the minority class; random upsampling of all classes to equal frequency of the majority class; and synthetic minority oversampling technique (SMOTE), an algorithm that upsamples by generation of novel data points through interpolation [3].

Each methodology was implemented using the `sampling` option of the function `train()` using the R package `caret`, v6.0-86 [4], except class weighting, which was assigned using the `weights` option. Model performances using each sampling methodology were then compared to our data thinning approach and an additional model using no resampling as a null comparison (S3 and S4 Tables).

**References**

1. He H, Garcia EA. Learning from Imbalanced Data. IEEE Trans Knowl Data Eng. 2009;21: 1263–1284. doi:10.1109/TKDE.2008.239

2. Rendón E, Alejo R, Castorena C, Isidro-Ortega FJ, Granda-Gutiérrez EE. Data Sampling Methods to Deal With the Big Data Multi-Class Imbalance Problem. Appl Sci. 2020;10: 1276. doi:10.3390/app10041276

3. Chawla NV, Bowyer KW, Hall LO, Kegelmeyer WP. SMOTE: Synthetic Minority Over-sampling Technique. J Artif Intell Res. 2002;16: 321–357. doi:10.1613/jair.953

4. Kuhn M. caret: Classification and Regression Training. R package version 6.0-86. 2020. Available: https://CRAN.R-project.org/package=caret
