# Supplementary material for: Predicting the animal hosts of coronaviruses from compositional biases of spike protein and whole genome sequences through machine learning
Source: PLoS Pathog. 2021 Apr 20;17(4):e1009149. doi: 10.1371/journal.ppat.1009149 (PMC8087038; doi:10.1371/journal.ppat.1009149)
Supplement: S1 Table — Descriptive statistics of number of sequences per coronavirus species or unranked subspecies (i.e., unique taxonomic ids) pre- and post-data thinning procedure to a maximum of 20 sequences per host-virus combination. Data shown separately for coronavirus spike proteins and whole genome sequences. (DOCX) [file ppat.1009149.s006.docx]

|  | **Spike protein dataset** | | **Whole genome dataset** | |
| --- | --- | --- | --- | --- |
|  | **Pre-data thinning** | **Post-data thinning** | **Pre-data thinning** | **Post-data thinning** |
| sd | 126.9 | 5.1 | 51.7 | 4.6 |
| range | 1, 1802 | 1, 28 | 1, 555 | 1, 23 |
| n (%) of species with > 20 sequences | 12 (5.4%) | | 10 (4.9%) | |
