# Supplementary material for: Predicting the animal hosts of coronaviruses from compositional biases of spike protein and whole genome sequences through machine learning
Source: PLoS Pathog. 2021 Apr 20;17(4):e1009149. doi: 10.1371/journal.ppat.1009149 (PMC8087038; doi:10.1371/journal.ppat.1009149)
Supplement: S2 Table — Number of genome sequences pre and post-data thinning procedure (to a maximum of 20 sequences per host-species combination) and number of coronavirus species or unranked subspecies (i.e., unique taxonomic ids) sourced from each host category. Data shown separately for coronavirus spike proteins and whole genome sequences. Note that although most coronaviruses were only known to infect a single host category, several coronaviruses infected multiple host categories and are represented across multiple counts of taxonomic ids. (DOCX) [file ppat.1009149.s007.docx]

|  | **Spike protein dataset** | | |
| --- | --- | --- | --- |
| **Host category** | **No. sequences (pre-data thinning)** | **No. sequences (post-data thinning)** | **No. coronaviruses** |
| bird | 527 | 84 | 24 |
| camelid | 323 | 63 | 7 |
| carnivore | 125 | 103 | 48 |
| human | 392 | 78 | 4 |
| rodent | 49 | 49 | 22 |
| swine | 2010 | 104 | 23 |
| yangochiroptera | 72 | 72 | 49 |
| yinpterochiroptera | 97 | 97 | 48 |
| **total** | 3595 | 650 | 222 |
|  | **Whole genome dataset** | | |
| bird | 363 | 66 | 23 |
| camelid | 305 | 58 | 7 |
| carnivore | 83 | 75 | 41 |
| human | 233 | 78 | 4 |
| rodent | 32 | 32 | 18 |
| swine | 688 | 91 | 22 |
| yangochiroptera | 45 | 45 | 33 |
| yinpterochiroptera | 66 | 66 | 39 |
| **total** | 1815 | 511 | 185 |
