# Supplementary material for: Predicting the animal hosts of coronaviruses from compositional biases of spike protein and whole genome sequences through machine learning
Source: PLoS Pathog. 2021 Apr 20;17(4):e1009149. doi: 10.1371/journal.ppat.1009149 (PMC8087038; doi:10.1371/journal.ppat.1009149)
Supplement: S4 Table — Model diagnostics describing overall performance using whole genome predictor features as in Table 2 when applying alternative sampling methodologies for class imbalance. Each methodology was applied to training data, retaining the same outer validation sets in all cases for comparability. CI denotes confidence interval, Kappa denotes Cohen’s Kappa statistic, mAUC denotes multiclass area-under-curve statistic, and F1macro denotes F1 score calculated using macro-averaging (performance on each host category weighted equally). (DOCX) [file ppat.1009149.s009.docx]

| **Sampling methodology** | **Accuracy (95% CI)** | **Kappa** | **mAUC** | **F1_macro_** |
| --- | --- | --- | --- | --- |
| data thinning  (as in Table 2) | 0.728 (0.687, 0.766) | 0.688 | 0.902 | 0.758 |
| class weighting | 0.703 (0.661, 0.742) | 0.660 | 0.891 | 0.737 |
| downsampling | 0.656 (0.613, 0.697) | 0.608 | 0.880 | 0.712 |
| upsampling | 0.710 (0.669, 0.749) | 0.669 | 0.894 | 0.744 |
| SMOTE | 0.496 (0.451, 0.541) | 0.410 | 0.816 | 0.544 |
| (none) | 0.734 (0.693, 0.772) | 0.695 | 0.911 | 0.764 |
