# Supplementary material for: Predicting the animal hosts of coronaviruses from compositional biases of spike protein and whole genome sequences through machine learning
Source: PLoS Pathog. 2021 Apr 20;17(4):e1009149. doi: 10.1371/journal.ppat.1009149 (PMC8087038; doi:10.1371/journal.ppat.1009149)
Supplement: S5 Table — Model diagnostics describing overall performance as in Table 2, repeating analyses for an additional ten random seeds. All metrics given represent the mean diagnostic with standard deviation in brackets. Kappa denotes Cohen’s Kappa statistic, mAUC denotes multiclass area-under-curve statistic, and F1macro denotes F1 score calculated using macro-averaging (performance on each host category weighted equally). (DOCX) [file ppat.1009149.s010.docx]

| **Predictor features** | **Accuracy** | **Kappa** | **mAUC** | **F1_macro_** |
| --- | --- | --- | --- | --- |
| Spike protein | 0.727 (0.007) | 0.687 (0.009) | 0.901 (0.006) | 0.758 (0.006) |
| Whole genome | 0.729 (0.004) | 0.690 (0.004) | 0.906 (0.005) | 0.758 (0.004) |
