# Supplementary material for: Predicting the animal hosts of coronaviruses from compositional biases of spike protein and whole genome sequences through machine learning
Source: PLoS Pathog. 2021 Apr 20;17(4):e1009149. doi: 10.1371/journal.ppat.1009149 (PMC8087038; doi:10.1371/journal.ppat.1009149)
Supplement: S6 Table — Model diagnostics describing overall performance when applied to predict host category of held-out coronaviruses. Balanced accuracy denotes 0.5*(sensitivity + specificity). (DOCX) [file ppat.1009149.s011.docx]

| **Host category** | **Balanced accuracy** | **Precision** | **Recall** | **F1 score** |
| --- | --- | --- | --- | --- |
| bird | 0.976 | 1.000 | 0.952 | 0.976 |
| camelid | 0.796 | 0.615 | 0.635 | 0.625 |
| carnivore | 0.932 | 0.852 | 0.893 | 0.872 |
| human | 0.613 | 0.857 | 0.231 | 0.364 |
| rodent | 0.951 | 0.818 | 0.918 | 0.865 |
| swine | 0.824 | 0.958 | 0.654 | 0.777 |
| yangochiroptera | 0.881 | 0.445 | 0.903 | 0.596 |
| yinpterochiroptera | 0.830 | 0.673 | 0.722 | 0.697 |
