# Supplementary material for: Predicting the animal hosts of coronaviruses from compositional biases of spike protein and whole genome sequences through machine learning
Source: PLoS Pathog. 2021 Apr 20;17(4):e1009149. doi: 10.1371/journal.ppat.1009149 (PMC8087038; doi:10.1371/journal.ppat.1009149)
Supplement: S7 Table — Model diagnostics describing overall performance when applied to predict host category of held-out coronaviruses. Balanced accuracy denotes 0.5*(sensitivity + specificity). (DOCX) [file ppat.1009149.s012.docx]

| **Host category** | **Balanced accuracy** | **Precision** | **Recall** | **F1 score** |
| --- | --- | --- | --- | --- |
| bird | 0.955 | 1.000 | 0.909 | 0.952 |
| camelid | 0.734 | 0.457 | 0.552 | 0.500 |
| carnivore | 0.918 | 0.969 | 0.840 | 0.900 |
| human | 0.730 | 0.760 | 0.487 | 0.594 |
| rodent | 0.935 | 0.933 | 0.875 | 0.903 |
| swine | 0.790 | 0.789 | 0.615 | 0.691 |
| yangochiroptera | 0.910 | 0.442 | 0.933 | 0.600 |
| yinpterochiroptera | 0.882 | 0.757 | 0.803 | 0.779 |
