# Supplementary material for: Predicting the animal hosts of coronaviruses from compositional biases of spike protein and whole genome sequences through machine learning
Source: PLoS Pathog. 2021 Apr 20;17(4):e1009149. doi: 10.1371/journal.ppat.1009149 (PMC8087038; doi:10.1371/journal.ppat.1009149)
Supplement: S8 Table — Model diagnostics describing overall performance using envelope protein predictor features as in Table 2. The same data thinning and cross-validation methodologies were applied as in Materials and Methods, thinning 1781 down to 458 envelope protein sequences from 144 coronaviruses. CI denotes confidence interval, Kappa denotes Cohen’s Kappa statistic, mAUC denotes multiclass area-under-curve statistic, and F1macro denotes F1 score calculated using macro-averaging (performance on each host category weighted equally). (DOCX) [file ppat.1009149.s013.docx]

| **Accuracy (95% CI)** | **Kappa** | **mAUC** | **F1_macro_** |
| --- | --- | --- | --- |
| 0.659 (0.614, 0.703) | 0.608 | 0.878 | 0.626 |
