# Supplementary figures and images for: Predicting the animal hosts of coronaviruses from compositional biases of spike protein and whole genome sequences through machine learning
Source: PLoS Pathog. 2021 Apr 20;17(4):e1009149. doi: 10.1371/journal.ppat.1009149 (PMC8087038; doi:10.1371/journal.ppat.1009149)

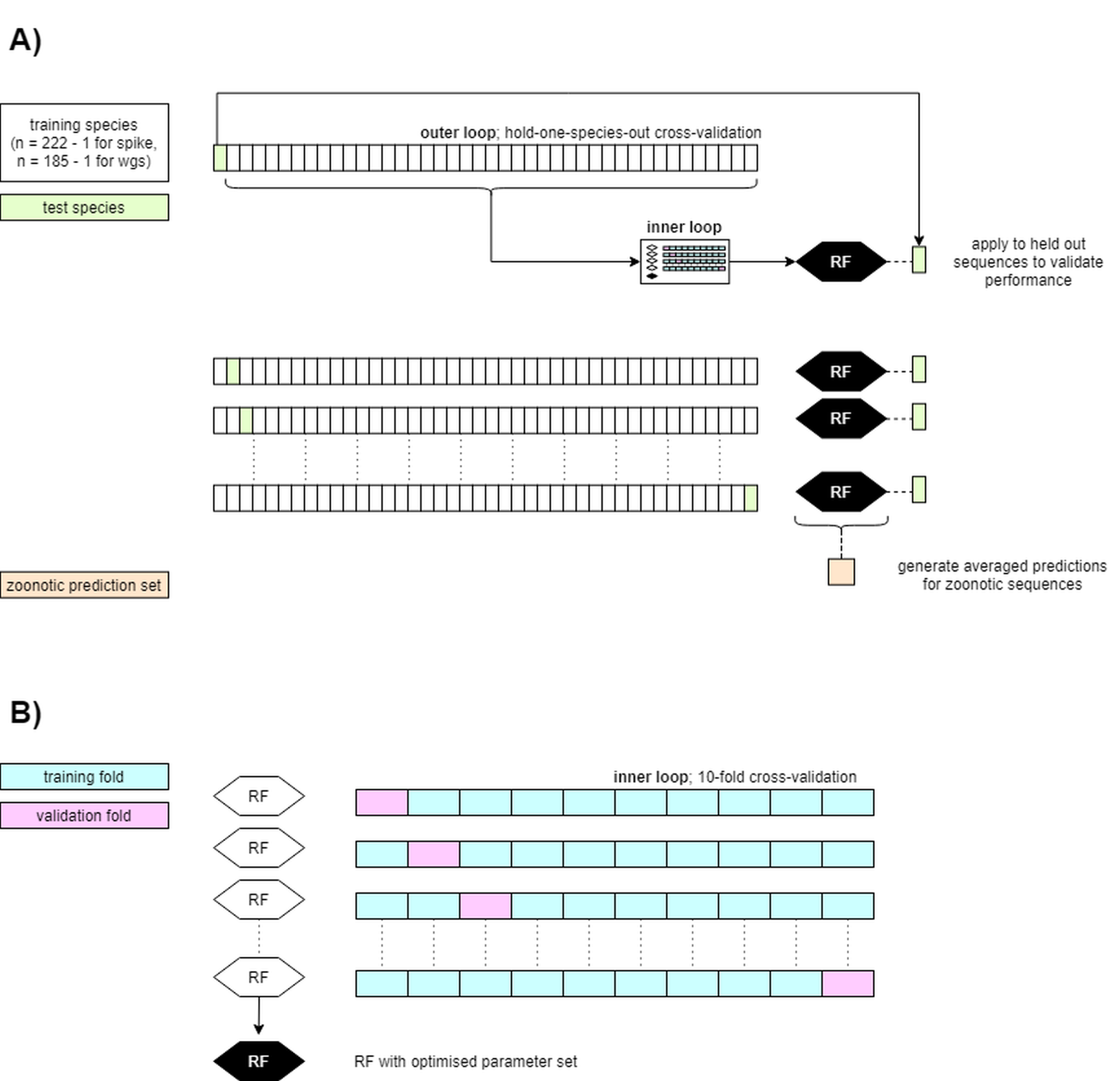

Supplement: S1 Fig — Data partitioning diagram indicating the machine learning procedure used, distinguishing A) the outer loop using hold-one-out cross-validation applied to coronavirus species or unranked subspecies (with the aim of validating model performance) from B) the inner loop using 10-fold cross-validation (with the aim of optimising model parameters). Distribution of outcome classes (host category) were preserved when sampling data folds during each inner loop. Zoonotic coronavirus sequences sampled from humans that were not used for model training are also distinguished. RF denotes a random forest model, while ‘spike’ and ‘wgs’ refer to spike protein feature dataset and whole genome feature dataset, respectively. (TIF) [file ppat.1009149.s014.tif]

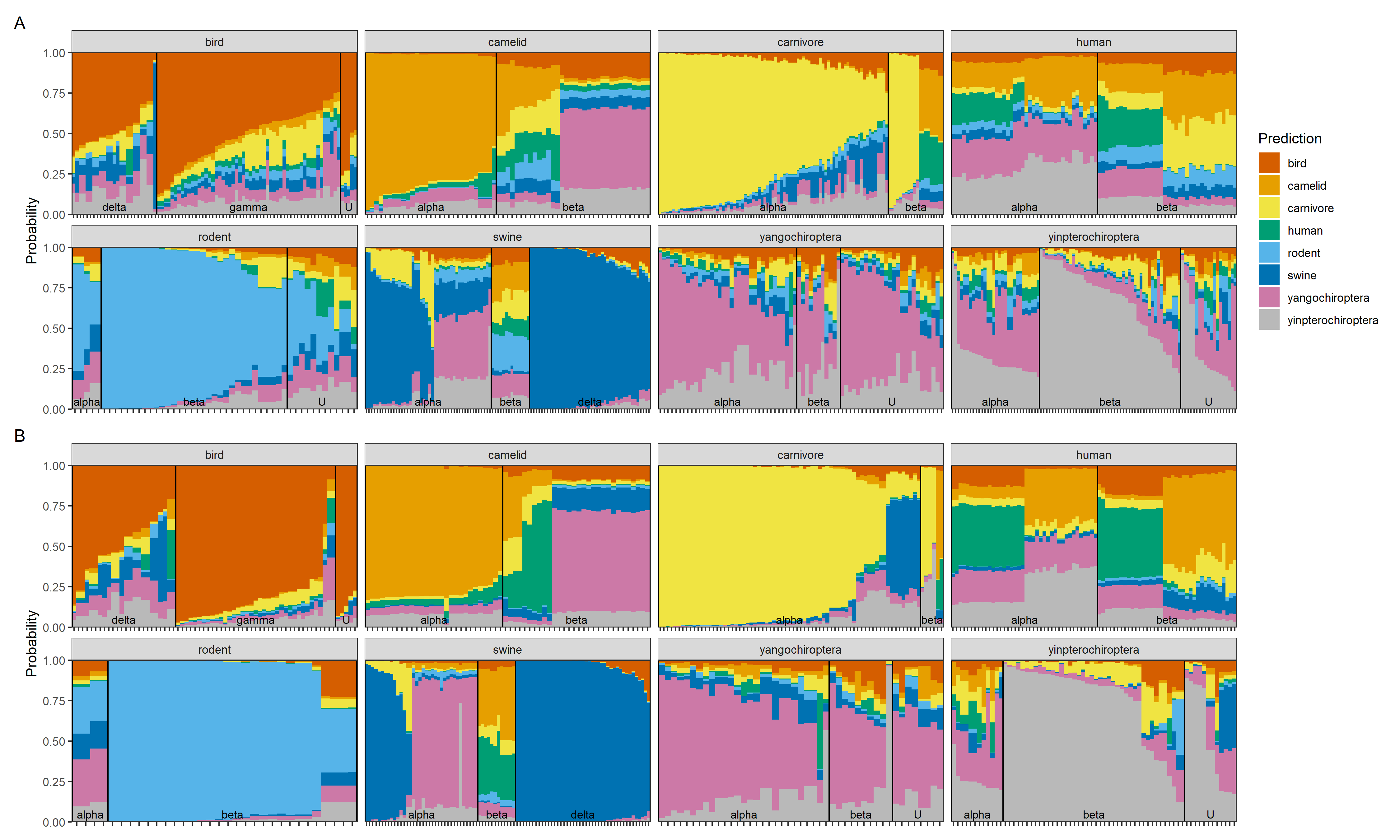

Supplement: S4 Fig — Stacked bar plots of predicted probabilities of each host category obtained from random forest models trained on A) spike protein and B) whole genome composition features as in Fig 3 when separated and ordered by genus (either Alphacoronavirus, Betacoronavirus, Gammacoronavirus, Deltacoronavirus, or “U” to indicated unassigned), with secondary ordering of largest to smallest probability of the correct host. (TIF) [file ppat.1009149.s017.tif]

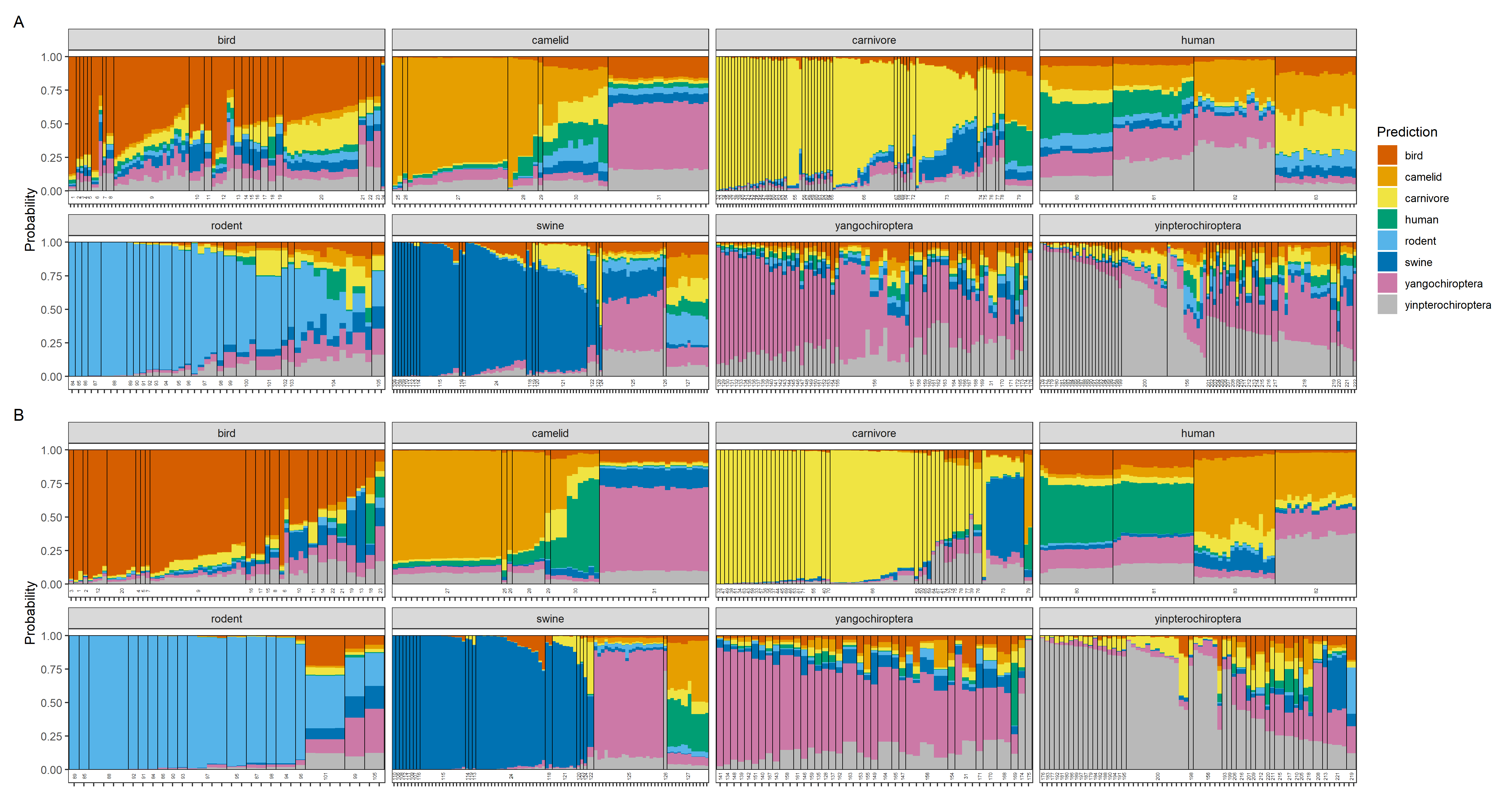

Supplement: S5 Fig — Stacked bar plots of predicted probabilities of each host category obtained from random forest models trained on A) spike protein and B) whole genome composition features as in Fig 3 when separated and ordered by species or unranked subspecies (i.e., unique taxonomic ids), with secondary ordering of largest to smallest probability of the correct host. See S1 Text for species key. (TIF) [file ppat.1009149.s018.tif]

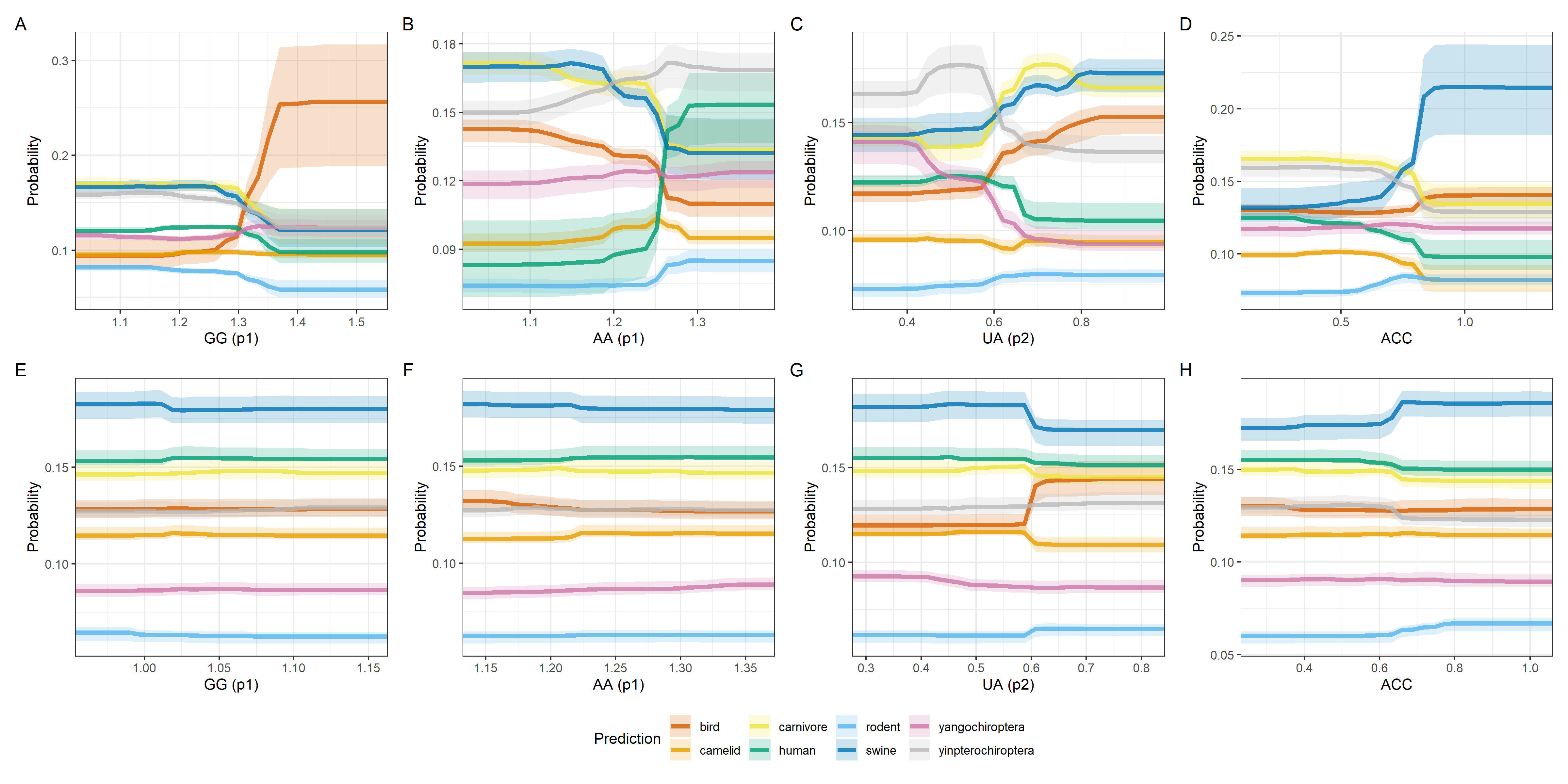

Supplement: S6 Fig — Model-predicted marginal probability of each coronavirus host category as functions of the four most informative genome composition bias features of spike protein sequences. A)–D) depict random forests trained on spike protein sequences and E)–H) depict probabilities as functions of the same features within random forests trained on whole genome sequences for comparison. Lines denote median values across A)–D) m = 225 and E)–H) m = 187 random forests during hold-one-out cross-validation. Shaded areas denote 2.5th and 97.5th percentiles. Colour key denotes host category. (TIF) [file ppat.1009149.s019.tif]

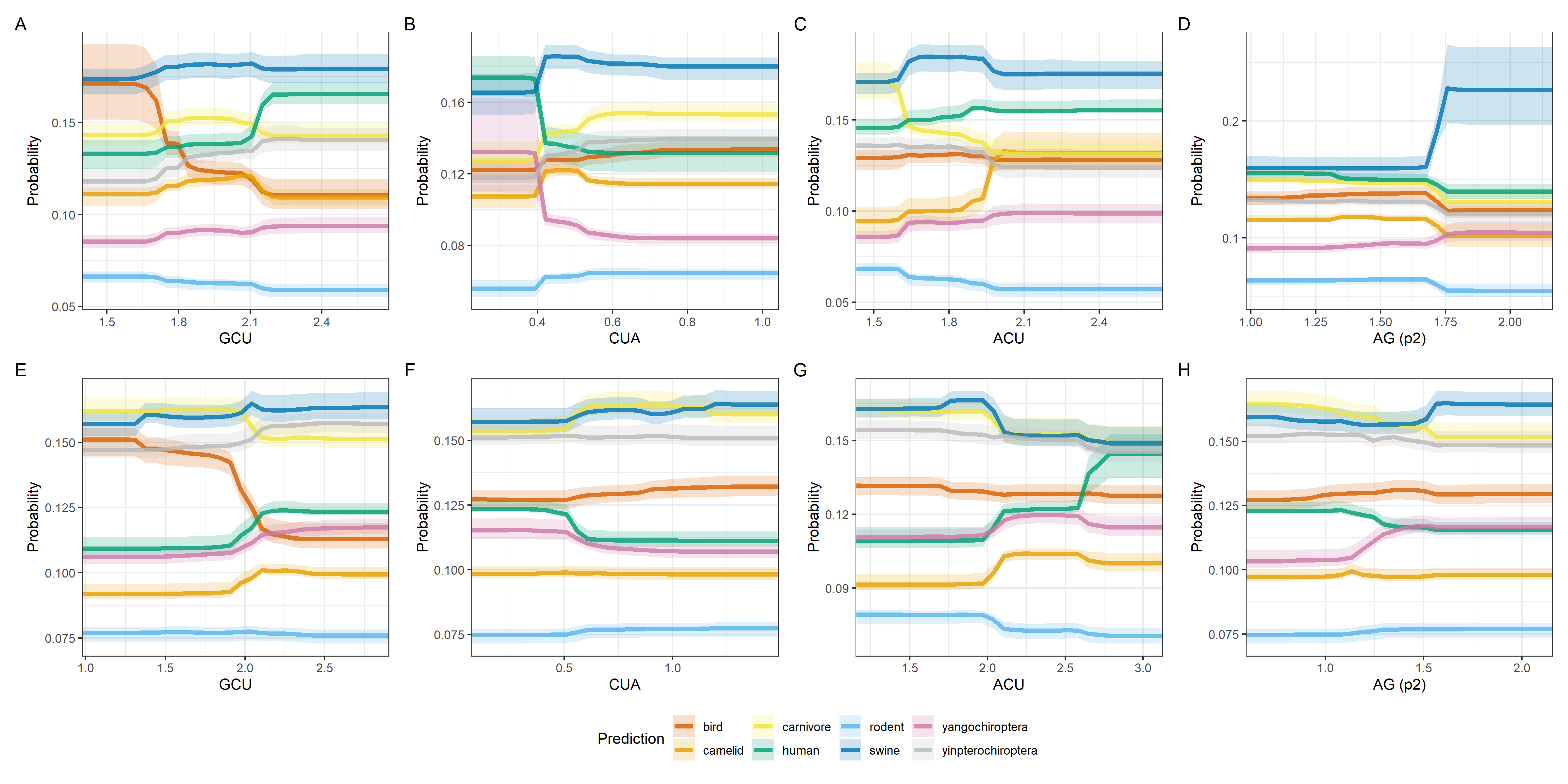

Supplement: S7 Fig — Model-predicted marginal probability of each coronavirus host category as functions of the four most informative genome composition bias features of whole genome sequences. A)–D) depict random forests trained on whole genome sequences and E)–H) depict probabilities as functions of the same features within random forests trained on spike protein sequences for comparison. Lines denote median values across A)–D) m = 187 and E)–H) m = 225 random forests during hold-one-out cross-validation. Shaded areas denote 2.5th and 97.5th percentiles. Colour key denotes host category. (TIF) [file ppat.1009149.s020.tif]
