# Supplementary material for: Predicting the animal hosts of coronaviruses from compositional biases of spike protein and whole genome sequences through machine learning
Source: PLoS Pathog. 2021 Apr 20;17(4):e1009149. doi: 10.1371/journal.ppat.1009149 (PMC8087038; doi:10.1371/journal.ppat.1009149)
Supplement: S1 Text — (DOCX) [file ppat.1009149.s021.docx]

1. Pheasant coronavirus

2. Falcon coronavirus UAE-HKU27

3. European turkey coronavirus 080385d

4. Pigeon coronavirus UAE-HKU29

5. Houbara coronavirus UAE-HKU28

6. Duck coronavirus

7. Bulbul coronavirus HKU11-796

8. White-eye coronavirus HKU16

9. Infectious bronchitis virus

10. Sparrow deltacoronavirus

11. Night heron coronavirus HKU19

12. Avian coronavirus

13. Sparrow coronavirus HKU17

14. Munia coronavirus HKU13-3514

15. Quail deltacoronavirus

16. Quail coronavirus UAE-HKU30

17. Bulbul coronavirus HKU11-934

18. Common moorhen coronavirus HKU21

19. Magpie-robin coronavirus HKU18

20. Turkey coronavirus

21. Thrush coronavirus HKU12-600

22. Wigeon coronavirus HKU20

23. Canada goose coronavirus

24. Porcine deltacoronavirus

25. Camel coronavirus HKU23

26. Alpaca respiratory coronavirus

27. Camel alphacoronavirus

28. Camel alphacoronavirus Camel229E

29. Bovine coronavirus isolate Alpaca

30. Dromedary camel coronavirus HKU23

31. Middle East respiratory syndrome-related coronavirus

32. Feline coronavirus UU2

33. Feline coronavirus UU11

34. Feline coronavirus UU10

35. Feline coronavirus UU9

36. Feline coronavirus UU19

37. Feline coronavirus UU31

38. Feline coronavirus UU18

39. SARS coronavirus civet020

40. Feline coronavirus UU54

41. SARS coronavirus B039

42. SARS coronavirus civet019

43. Feline coronavirus UU22

44. Feline coronavirus UU7

45. Feline coronavirus UU8

46. Feline coronavirus UU20

47. Feline coronavirus UU3

48. SARS coronavirus CS21

49. Feline coronavirus UU23

50. Feline coronavirus RM

51. Feline coronavirus UU15

52. Feline alphacoronavirus 1

53. Feline coronavirus UU30

54. SARS coronavirus ES260

55. Feline infectious peritonitis virus

56. SARS coronavirus CS24

57. Feline coronavirus UU34

58. Feline coronavirus UU40

59. Feline coronavirus UU4

60. SARS coronavirus ES191

61. Feline coronavirus UU47

62. SARS coronavirus civet014

63. Feline coronavirus UU21

64. SARS coronavirus civet010

65. Feline coronavirus UU17

66. Feline coronavirus

67. Civet SARS CoV 007/2004

68. SARS coronavirus WF188

69. Feline coronavirus UU5

70. Feline coronavirus UU24

71. Feline coronavirus UU16

72. Ferret enteric coronavirus

73. Canine coronavirus

74. Ferret systemic coronavirus

75. Mink coronavirus strain WD1133

76. Ferret coronavirus

77. Alphacoronavirus Mink/China/1/2016

78. Mink coronavirus strain WD1127

79. Canine respiratory coronavirus

80. Human coronavirus HKU1

81. Human coronavirus NL63

82. Human coronavirus 229E

83. Human coronavirus OC43

84. Murine coronavirus repJHM/RA59

85. Murine coronavirus RA59/R13

86. Murine coronavirus SA59/RJHM

87. Murine hepatitis virus strain A59

88. Murine coronavirus

89. Murine coronavirus inf-MHV-A59

90. Murine coronavirus RA59/SJHM

91. Murine coronavirus MHV-JHM.IA

92. Murine coronavirus RJHM/A

93. Murine coronavirus repA59/RJHM

94. Rat coronavirus Parker

95. Rat coronavirus

96. Murine coronavirus MHV-3

97. Murine hepatitis virus

98. Murine coronavirus MHV-1

99. Lucheng Rn rat coronavirus

100. Longquan Rl rat coronavirus

101. Betacoronavirus HKU24

102. Longquan Aa mouse coronavirus

103. Alphacoronavirus UKRn3

104. Rodent coronavirus

105. Coronavirus AcCoV-JC34

106. Deltacoronavirus PDCoV/USA/Illinois133/2014

107. Porcine deltacoronavirus KNU14-04

108. Deltacoronavirus PDCoV/USA/Illinois134/2014

109. Porcine deltacoronavirus 8734/USA-IA/2014

110. Deltacoronavirus PDCoV/USA/Illinois121/2014

111. Deltacoronavirus PDCoV/USA/Illinois136/2014

112. Deltacoronavirus PDCoV/USA/Ohio137/2014

113. TGEV Miller M6

114. TGEV Purdue P115

115. Porcine coronavirus HKU15

116. Swine deltacoronavirus OhioCVM1/2014

117. Porcine deltacoronavirus Sichuan

118. Coronavirus HKU15

119. TGEV virulent Purdue

120. TGEV Miller M60

121. Transmissible gastroenteritis virus

122. Swine enteric coronavirus

123. Porcine respiratory coronavirus

124. PRCV ISU-1

125. Porcine epidemic diarrhea virus

126. Rhinolophus bat coronavirus HKU2

127. Porcine hemagglutinating encephalomyelitis virus

128. BtMf-AlphaCoV/JX2012

129. BtMf-AlphaCoV/GD2012-a

130. Bat coronavirus MfulBtCoV/3759-1

131. BtMf-AlphaCoV/HuB2013-a

132. Bat coronavirus MfulBtCoV/3709

133. Bat coronavirus MsBtCoV/4068

134. BtMf-AlphaCoV/HeN2013

135. BtMf-AlphaCoV/AH2011

136. Bat coronavirus MfulBtCoV/3736-1

137. Miniopterus schreibersii bat coronavirus 1-related

138. Bat coronavirus MsBtCoV/4056

139. Coronavirus BtSk-AlphaCoV/GX2018C

140. BtMf-AlphaCoV/GD2012

141. BtMf-AlphaCoV/FJ2012

142. Coronavirus BtSk-AlphaCoV/GX2018B

143. Miniopterus pusillus bat coronavirus HKU8-related

144. Bat coroanvirus MsBtCoV/4001-1

145. BtMf-AlphaCoV/GD2012-b

146. BtVs-BetaCoV/SC2013

147. Tylonycteris pachypus bat coronavirus HKU4-related

148. Coronavirus BtSk-AlphaCoV/GX2018A

149. Alphacoronavirus Bat-CoV/P.kuhlii/Italy/206645-41/2011

150. Bat coronavirus MsBtCoV/3710

151. BtMf-AlphaCoV/HuB2013

152. Scotophilus kuhlii coronavirus

153. BtPa-BetaCoV/GD2013

154. BtTp-BetaCoV/GX2012

155. Coronavirus Neoromicia/PML-PHE1/RSA/2011

156. Bat coronavirus

157. Miniopterus bat coronavirus/Kenya/KY33/2006

158. Alphacoronavirus Bat-CoV/P.kuhlii/Italy/3398-19/2015

159. Scotophilus kuhlii bat coronavirus 512-related

160. Chaerephon bat coronavirus/Kenya/KY22/2006

161. Alphacoronavirus Bat-CoV/P.kuhlii/Italy/206679-3/2010

162. Pipistrellus abramus bat coronavirus HKU5-related

163. BtNv-AlphaCoV/SC2013

164. Bat coronavirus CDPHE15/USA/2006

165. Myotis lucifugus coronavirus

166. Chaerephon bat coronavirus/Kenya/KY41/2006

167. Coronavirus BtSk-AlphaCoV/GX2018D

168. Hypsugo bat coronavirus HKU25

169. Tylonycteris bat coronavirus HKU33

170. BtMr-AlphaCoV/SAX2011

171. Bat alphacoronavirus

172. Miniopterus bat coronavirus/Kenya/KY27/2006

173. Betacoronavirus BtCoV/KW2E-F93/Nyc_spec/GHA/2010

174. BtMs-AlphaCoV/GS2013

175. Bat coronavirus Cp/Yunnan2011

176. BtRf-BetaCoV/HeB2013

177. Coronavirus BtRs-BetaCoV/YN2018C

178. BtRf-BetaCoV/HeN2013

179. Bat SARS CoV Rf1/2004

180. Bat SARS-like coronavirus WIV1

181. BtRf-BetaCoV/SX2013

182. SARS coronavirus Rs_672/2006

183. Bat SARS-like coronavirus Rs3367

184. Coronavirus BtRs-BetaCoV/YN2018A

185. BtRf-BetaCoV/HuB2013

186. Bat SARS-like coronavirus YNLF_31C

187. SARS-like coronavirus WIV16

188. BtRs-BetaCoV/YN2013

189. Coronavirus BtRs-BetaCoV/YN2018D

190. BtRf-BetaCoV/JL2012

191. BtRs-BetaCoV/GX2013

192. Bat SARS-like coronavirus RsSHC014

193. Bat coronavirus Rp/Shaanxi2011

194. Coronavirus BtRs-BetaCoV/YN2018B

195. Bat SARS CoV Rm1/2004

196. Bat SARS Cov Rs806/2006

197. Bat SARS-like coronavirus YNLF_34C

198. BtRs-BetaCoV/HuB2013

199. Coronavirus BtRl-BetaCoV/SC2018

200. Bat SARS-like coronavirus

201. Bat coronavirus RaTG13

202. Eidolon bat coronavirus/Kenya/KY24/2006

203. Rousettus bat coronavirus/Kenya/KY06/2006

204. SARS-like coronavirus BatCoV/BB9904/BGR/2008

205. Rousettus bat coronavirus HKU9

206. Rousettus aegyptiacus bat coronavirus 229E-related

207. Bat coronavirus Shandong/977/2006

208. Rhinolophus bat coronavirus HKU32

209. Severe acute respiratory syndrome-related coronavirus

210. Hipposideros pomona bat coronavirus CHB25

211. Hipposideros pomona bat coronavirus HKU10-related

212. Bat Hp-betacoronavirus/Zhejiang2013

213. Coronavirus BtRs-AlphaCoV/YN2018

214. Cardioderma bat coronavirus/Kenya/KY43/2006

215. BtRf-AlphaCoV/HuB2013

216. Bat coronavirus BM48-31/BGR/2008

217. BtRf-AlphaCoV/YN2012

218. 229E-related bat coronavirus

219. Rousettus bat coronavirus

220. Coronavirus BtRt-BetaCoV/GX2018

221. Swine acute diarrhea syndrome related coronavirus

222. Bat coronavirus RsBtCoV/3716
