# Supplementary material for: Predicting the animal hosts of coronaviruses from compositional biases of spike protein and whole genome sequences through machine learning
Source: PLoS Pathog. 2021 Apr 20;17(4):e1009149. doi: 10.1371/journal.ppat.1009149 (PMC8087038; doi:10.1371/journal.ppat.1009149)
Supplement: Attachment — Submitted filename: Response_to_review_letter.docx [file ppat.1009149.s022.docx]

| PLOS Pathogens Editorial Team  PLOS  1265 Battery Street Floor 2 San Francisco, CA 94111 United States |  | Department of Biostatistics  Block F Waterhouse Building  University of Liverpool  1-5 Brownlow Street  Liverpool  L69 3GL  United Kingdom  Email: liam.brierley@liverpool.ac.uk  Tel: +44 (0) 151 7949711 |
| --- | --- | --- |
| 5 March 2021 |  |  |

Dear PLOS Pathogens Editorial Team,

Re: **Predicting animal hosts of coronaviruses from compositional biases of spike protein and whole genome sequences through machine learning**

We would like to thank the reviewers for their close attention to the details and scope of our work throughout. We feel that their comments have really helped to strengthen and clarify the paper and hope we’ve addressed them all. The main improvements include some additional results describing model predictive performance using several alternative methodologies, as well as clarification and better support given for methodological decisions including our original undersampling procedure (now referred to as “data thinning”). We demonstrate only minor variability arising from alternative approaches, and therefore our main models presented in the manuscript are retained. Please find detailed responses to the reviewer comments copied in below in blue, where R refers to reviewer number, MC refers to major comment number, and L refers to lines in the revised manuscript unless stated otherwise.

Yours sincerely,

Liam Brierley

[liam.brierley@liverpool.ac.uk](mailto:liam.brierley@liverpool.ac.uk)

Reviewer's Responses to Questions

**Part I - Summary**
Please use this section to discuss strengths/weaknesses of study, novelty/significance, general execution and scholarship.

Reviewer #1: The authors analyze spike protein and whole genomes of various coronaviruses using measures such as dinucleotide and codon usage biases to predict animal host using random forests. The approach appears to be surprisingly successful given the relatively simple measures used. Such an approach may be useful in determining genomic biases that are preferred by various coronaviruses as they adapt to their respective hosts.

Reviewer #2: The paper uses the compositional signatures of various coronaviruses to predict hosts with the aim of identifying the animal host of SARS-CoV-2. To do so, the authors use the random-forest machine-learning algorithm for classification, followed by analysis of the importance of each genomic feature.

While the development of algorithms for host prediction are extremely important (the current coronavirus emergence scenario is a good example), my general opinion is that such methods have to be transparent in their predictive mechanism. A black box approach, as used in the current study is potentially susceptible to a range of artefacts, of which over-fitting of compositional features through the underlying clustering of sequences by phylogeny relationships represents a substantial underlying compounding factor.

Although we accept these methods are unavoidably prone to confounding by phylogenetic relatedness and that machine learning methods are often treated as black box approaches, we are able to account for some phylogenetic relatedness through our validation approach and to describe predictive mechanisms through our use of random forests. More details are provided in the response to comments below, but in particular see R1MC5 and R2MC2. Predictive mechanisms are made challenging to conceptualise because our machine learning method is an aggregation of many individually weak predictors – but this is not impossible. We offer transparent example profiles of the most important of these individual predictors in Supplementary Figures S6 & S7.

As I see it, (although I would be interested to hear the authors’ opinion on this), the basic problem is the compositional similarities used in the algorithm, such as codon choice and dinucleotides that specify amino acids, can also originate simply from virus sequences being similar to each other through genetic relatedness. Assignment of host based on compositional features might then simply represent a match to the phylogenetic clusters of viruses infecting that particular host. Indeed, although the figure is unlabelled, genus assignments seem to correlate with their host predictions in Fig. 2. Predicted hosts of pig coronaviruses (delta, alfa and beta), might correspond to the three blocks in host prediction. The alfa and bertacoronavirus blocks (on the right) furthermore look remarkably similar to the patterns of the human alfa and beta human seasonal human coronavirus. Similarly for camel viruses, where the first block might be 229E (Alfacoronavirus) and the second MERS-CoV-2-related (Betacoronavirus) based on the relative numbers of camel viruses provided in Table S1. At the very least the figure should be annotated to provide the genus assignments to demonstrate the extent to which these contribute to host prediction patterns.

We agree that similarity in genome composition can simply be reflective of phylogenetic relatedness rather than evolutionary convergence (addressed in Discussion L393-402). In our approach, we attempt to minimise this confounding and control for relatedness of sequences within-virus via our outer hold-one-out cross-validation procedure, which removes all sequences from the same species. Further detail has been added to the results to emphasise this (see R1MC5).

We now provide labelled versions of Figure 2 depicting machine learning model predictions of hosts that group predictions by genus (Supplementary Figure S4) and by species or unranked subspecies (Supplementary Figure S5). We note that “blocking” of predictions is somewhat homogeneous within species, but less homogenous within genera.

For example, on the specific patterns discussed above, within the three “blocks” of similar predictions for pig coronaviruses, betacoronaviruses represent the unresolved block, though both deltacoronaviruses and alphacoronaviruses represent the majority-swine prediction block. This betacoronavirus block is somewhat similar to (but has different dominant predicted host groups than) the predictions for human betacoronaviruses HKU1 and OC43. Regarding predictions for camel coronaviruses, the large block of Yangochiroptera-dominated predictions are betacoronaviruses, though are not the only betacoronaviruses represented.

This demonstrates that predictions are not driven by phylogenetic similarity within-genus; instead, highly similar predictions result from within-species similarity in genomic biases, indicating a need to adjust at this taxonomic level in our hold-one-species-out cross-validation method.

In relation to that, I think it is a bit disingenuous to claim that MERS-CoV-2 was excluded from the training (lines 246-248) since the camel betacoronaviruses are very similar to MERS-CoV found in humans and it is therefore no surprise that the latter are then found to have a predicted camel origin (Fig. 3).

We exclude zoonotic, i.e., human-sourced sequences of MERS-CoV, though retain MERS-CoV-related camel viruses within training. In doing so we use these zoonotic viruses as a test case of ecological and public health interest, noting as above that these predictions could be compared to inference obtainable from a phylogeny (which would equally use such camel MERS-CoV sequences as input).

In terms of informative features, the observation that biases in codon positions 1 and 2 were the most predictive of host (with GG being the highest rank) might simply follow differences in amino acid composition between sequences that co-associated with genus.

While there is some clustering within-genus of codon bias values features used to train models (Figure 1) and we have added some quantification of this in Normalised Mutual Information values (see R1MC6), as in our comment above, this does not result in absolute clustering within-genus of model predictions during cross-validation.

We make no assumptions about the molecular mechanisms connecting dinucleotide and codon biases to host adaptation, which could feasibly involve an intermediate step of amino acid composition as discussed in the manuscript (Discussion L411-413). We argue that codon usage biases are of interest as features that give predictive resolution beyond genera, agnostic of mechanisms.

**Part II – Major Issues: Key Experiments Required for Acceptance**
Please use this section to detail the key new experiments or modifications of existing experiments that should be absolutely required to validate study conclusions.

Generally, there should be no more than 3 such required experiments or major modifications for a "Major Revision" recommendation. If more than 3 experiments are necessary to validate the study conclusions, then you are encouraged to recommend "Reject".

Reviewer #1: -The authors consider the spike protein as a special case but it may be that other proteins such as orf1ab or the RdRp may yield better results. At the very least some discussion should be added as to what would be expected if other gene(s) were analyzed. Also, a more detailed description of spike protein variation in the introduction would improve the justification for focusing on spike. The current description is fairly minimal.

We focus on the spike as a protein directly and extensively involved in molecular virus-host interactions. We have added further detail to the introduction about spike protein variability and selection (L60-62: “As such, this protein exhibits high variation..”; L68-73: “Several methods..”).

We considered a comparison to alternative genes or proteins, though we note the orf1ab represents approximately 70% of the whole coronavirus genome and so we would not expect this to yield much different results to using whole genome sequences. Additionally, as a polyprotein product, most viral GenBank records do not have labelled nucleotide sequences corresponding to the individual RdRp protein.

Instead, we also include performance results from an additional model trained on composition of the envelope gene, which achieves some predictive accuracy but under-performs in all metrics compared to models trained on spike proteins and whole genomes (Supplementary Table S8). We have also expanded the discussion section on this point (L342-347: “The same evolutionary signatures would not be expected..”).

-There are 116 genomic features used for the ML dataset. Can these be explicitly listed somewhere, e.g. in a supplementary table?

Further information has been added in the Methods (L149: “4 nucleotide biases, 16 * 3 dinucleotide biases, 64 codon biases”), the features correspond to bias measurements for each possible nucleotide, dinucleotide and codon. Raw data files listing all features have also been additionally supplied with the code in a GitHub repository (https://github.com/lbrierley/cov_genome)

-The under-sampling strategy leads to a dramatic reduction in data utilized. Might it be possible to avoid this by instead introducing class weights during training?

Our approach in sampling a limited number of sequences belonging to the same virus species or unranked subspecies is motivated by the biased representation of viruses among available data and the high similarity of sequences within-species. Several virus species are extremely overrepresented among available sequences and if retained resulting validation metrics would be heavily weighted towards the classification of these frequently-sampled species. This is a distinct issue from biased composition of outcome classes (host categories).

We have added a new Supplementary Table S1 to emphasise the skewed distribution of available sequences per virus. We have also altered the terminology of our approach (now described as 'data thinning' throughout) and rewritten our Methods section (L170-177, paragraph starting “These predictive modelling methods..”) to distance this from a class imbalance-motivated undersampling.

However, we note that our approach does have a secondary effect of reducing existing class imbalances (Supplementary Table S2), and we also formally compare model performances when using imbalance-adjustment methods including class weighting, finding our approach gives equal or superior performance (Supplementary Methods S1, Supplementary Tables S3 & S4, see also R2MC7).

-As far as host categories were concerned, did the model allow for the possibility of same virus but multiple hosts? Multiple bats, for example, may be able to harbor the same virus.

As each individual data point in our analysis represents an individual virus sequence, by definition these can only have been sampled from a single host species. In a minority of cases, our data includes multiple sequences of the same virus species that were sampled from different host categories (e.g., MERS-CoV sequences sampled from bats and from camels). However, cross-species transmission within-host category (e.g., one bat species to another bat species) are not inferred at this scale of analysis.

-The inner/outer loop cross validation strategy is unusual and makes it unclear whether the train and test sets are really independent. If they are not independent, the results may be marred by overfitting. How different are the results if only one or the other of these strategies (leave-one-out or 10-fold cross-validation) were used?

We use both inner and outer loops of cross-validation for specific functions. The inner loop functions as a more ‘traditional’ method of optimising chosen parameters when constructing random forest models, whereas the purpose of the outer loop is to specifically generate validation sets that do not suffer from phylogenetic nonindependence with training sets by excluding all sequences from a given coronavirus (species or unranked subspecies) in each instance. We have expanded the relevant section of the methods to better explain this (L193-203: “As genome composition is highly homogenous..”) and additionally replaced the previous Supplementary Figure S1 with an entirely new figure that better illustrates the data partitioning used in both inner and outer cross-validation loops. As each of these loops of cross-validation fulfils a different purpose, we do not believe models excluding either would be meaningful (not using the inner loop would require potentially suboptimal model parameters to be chosen a priori, not using the outer loop would result in highly similar sequences being present in both training and test sets).

-The authors compare their "spike protein" and "whole genome" models at least twice: in the second paragraph of results ("Hierarchical clustering based on RSCU 182values suggested codon usage was less distinct between genera within spike protein sequences") and then in the discussion ("which may explain the stronger prediction of carnivore hosts when using spike protein sequences''). However, in neither case they provide any statistical support for their statements. Are these differences statistically significant? Similarly, the statement “Among our dataset of 225 coronaviruses, we observed A- and U-ending codons to be overrepresented and G- and C- ending codons to be underrepresented” in the discussion is not analyzed statistically.

We have now supported these statements about within-genus clustering of codon usage and model-predicted probability of carnivore hosts for SARS-CoV with normalised mutual information values (L232-233) and mean/SD respectively (L371). We have also conducted one-sample tests to support statements about over and underrepresentation of codons within the Results (L227-230). Text been added to the Methods section describing our use of these calculations and tests (L156-163: “To assess over-or underrepresentation..”).

Reviewer #2: 1. There is no published source code for this analysis, rendering it unable to be completely reproduced. At the minimum, all code used to generate the results and figures should be published.

A link to the source code and supporting data has now been added to the Methods (https://github.com/lbrierley/cov_genome).

2. The paper does not make clear the rationale behind using random forests for modelling the hosts of coronaviruses. Given the class imbalance was resolved with under-sampling, other statistical classification approaches such as support vector machines, logistic regression, and k-nearest neighbors may also be useful. Indeed, some of these approaches (such as k-NN) are robust to class imbalance in the first place and no explanation for their exclusion is provided in the paper. A fuller comparison of the methods and their relative performances would strengthen the methodological advances claimed in the manuscript.

As in R1MC3 (see also R2MC7), negligible class imbalance remains after our data thinning procedure, and so class imbalance has no bearing on or methodology choice. We apply random forests for two primary reasons.

Firstly, as a rule-based learner (cf. instance-based learners such as kNN) they handle interaction between features and offer additional interpretability of feature-outcome relationships [1] – specifically, in informativeness via variable importance which we plot in Figure 4 and directionality via partial dependence, which we plot in Supplementary Figures 6 & 7. Our measures from both these plots are important for inference of the evolutionary dynamics of genome composition relative to host type.

Secondly, random forests natively handle multiclass classification problems. We note that logistic regression and support vector machines are intended for binary classification and use of these for our eight host categories would require repeating a series of one-vs-rest or one-vs-one classifications [2], adding another layer of uncertainty (and computing time) to the analysis.

We have clarified our reasoning for selecting random forests in the Methods section (L167-169: “We selected random forests..”)

3. The description of the training method is not fully clear and would be served by further explanation. For example, the reason the parameters were optimized within the inner loop is not fully explained within the main manuscript.

As in our response to R1MC5, we have now redrafted the relevant Methods text describing our inner and outer cross-validation procedures, supported by a new Supplementary Figure S1.

4. The paper is unclear whether dinucleotide frequencies, RSCU, and ENc were computed using a package and, if so, which package.

Details of the packages used have been added to the Methods (L163-165).

5. In relation to reproducibility, the manuscript does not describe whether a fixed random seed was used for the analysis and chosen beforehand. Doing so with the code supplied (see comment 1 above) would allow reproduction of the exact results presented as well as examination of the method over a variety of random seeds to ascertain its general performance.

As in R2MC1, code has been made available. We have clarified in the Methods that a fixed random seed was used (L214: “All analyses were initiated from a fixed random seed chosen a priori..”), and we have additionally repeated our main analyses using ten further random seeds, demonstrating little variability in model performance over different seeds (Supplementary Table S5).

6. Similarly, it is not explained in the paper how many times the analyses were run. In the absence of such information and reproducible source code, multiple testing can be a concern for the validity of the results.

As in R2MC1, code has been made available. This uses the random seed for the analytical run presented in the manuscript, noting that computational time restricts extensive repetition of all analyses over multiple seeds. We construct and validate a random forest for each held-out coronavirus species or unranked subspecies (n = 222 or 185 for spike protein or whole genome analysis; Supplementary Figure S1A) and each forest contains 1000 independent classification tree models (Methods L213: “All random forests were constructed using 1000 trees…”)

7. The paper does not fully describe on the rationale for selecting twenty random samples per host category per virus for under-sampling. There are methods for under-sampling based on selecting nearest neighbors that can maintain or improve performance while also reducing class imbalance. Examples of such methods are near miss and condensed nearest neighbor under-sampling.

As in our response to R1MC3, we now distinguish our data thinning approach to adjust for biased viral sequence availability from undersampling approaches adjusting for class imbalance.

This however has a secondary effect of reducing, but not eliminating class imbalance (Supplementary Table S2), and we therefore compared our approach to methods for class imbalance including downsampling, upsampling, class weighting and SMOTE (an algorithm similar to those suggested by the reviewer), based on available implementations in the model functions and packages chosen (Supplementary Methods S1).

We find that on a fixed validation set, our approach results in predictive performance comparable (or superior in the case of downsampling and SMOTE) to that of class imbalance adjustment methods (Supplementary Tables S3 & S4). We note that no adjustment also performs reasonably well, suggesting insensitivity to class imbalance for this particular dataset and modelling pipeline.

The choice of twenty viruses per host category per viruses is a heuristic one, intended to thin overrepresented species to numbers equivalent to the ~5^th^ percentile (new Supplemental Table S1, also Methods L174-177). However, we would argue that as genomic biases and host predictions were relatively homogeneous within species (Supplementary Figure S5, see also response to Reviewer 2’s Part I comments), altering this value to is likely to have little impact on model performance.

**Part III – Minor Issues: Editorial and Data Presentation Modifications**
Please use this section for editorial suggestions as well as relatively minor modifications of existing data that would enhance clarity.

Reviewer #1: -Typo on line 157: should read “rather than”.

This has now been corrected.

Reviewer #2: Lines 36, 39. Why are these called intermediate hosts?

We use intermediate host here as a well-accepted term for a host that is not itself a reservoir (i.e., it does not maintain a naturalised cycle of infection) but has acted as a step in transmission chains from animal reservoirs to humans [3,4].

**References:**

1.	Hastie T, Tibshirani R, Friedman J. Random Forests. In: Hastie T, Tibshirani R, Friedman J, editors. The Elements of Statistical Learning: Data Mining, Inference, and Prediction. New York, NY: Springer; 2009. pp. 587–604. doi:10.1007/978-0-387-84858-7_15

2.	James G, Witten D, Hastie T, Tibshirani R. Support Vector Machines. In: James G, Witten D, Hastie T, Tibshirani R, editors. An Introduction to Statistical Learning: with Applications in R. New York, NY: Springer; 2013. pp. 337–372. doi:10.1007/978-1-4614-7138-7_9

3.	Lloyd-Smith JO, Funk S, McLean AR, Riley S, Wood JLN. Nine challenges in modelling the emergence of novel pathogens. Epidemics. 2015;10: 35–39. doi:10.1016/j.epidem.2014.09.002

4.	Royce K, Fu F. Mathematically modeling spillovers of an emerging infectious zoonosis with an intermediate host. PLOS ONE. 2020;15: e0237780. doi:10.1371/journal.pone.0237780
